# 

**Published:** 1915-01-09

**Authors:** 


### Gifts and Bequests.

The King has sent a gift of pheasants to the Royal Hospital for Incurables, Putney Heath, of which institution His Majesty is patron.

Mr. Josiah Blaiberg, of Belsize Avenue, Hampstead, N.W., financial agent, left £50 to the infirmary (King Edward VII.'s Hospital), Cardiff, and numerous other charitable bequests.

After leaving numerous large bequests to Church and Mission charities, Mr. William Long, J.P., of Clarence Street, Gloucester, has made the Gloucester Royal Infirmary his residuary legatee. During his lifetime he gave to the Gloucester Provident Dispensary, the Magdalen Asylum, and the District Nursing Society the premises occupied by them.

St. Margaret's Works, Leicester, the largest community of workers in the borough, have allocated their collection for 1914, amounting to £914 6s. 9d., as follows: Leicester Royal Infirmary and Children's Hospital, £550; the War Funds, £274 2s. 9d.; the Prince of Wales's National Relief Fund, £121 17s. 9d.; the Belgian National Fund, £50 10s.; the Belgian Refugee Fund, £20 10s.; Tommy Atkins's Christmas Present Fund, £34 13s.; Princess Mary's Fund, £2 2s.; and £44 10s. has been granted to workpeople in departments of the works affected by the war.

### Personalia.

### EFFECTS OF THE WAR ON AN INFIRMARY STAFF.

A return presented to the Lambeth Board of Guardians shows how seriously a London infirmary has been affected by the war. The staff that has joined the Colours from this one institution alone consists of the following: Dr. G. F. Stebbing, first assistant medical officer, naval surgeon; S. Croxson, steward's senior clerk, private; H. T. Roffey, steward's second clerk, private; F. G. Read, male nurse and night mental attendant, private; H. J. Montford, relief man, able seaman; W. L. Payne, night receiving wardsman, gunner R.H.A.; D. Hodsdon, night watchman, private National Reserve; R. Dalton, stoker, able seaman; and J. Schween, laundry porter, lance-corporal. In addition, the board have lost the services of Dr. R. J. W. Oswald, district medical officer, who is a major in the R.A.M.C., and of Mr. F. W. H. Selby, superintendent relieving officer, now a sergeant in Kitchener's Army.

### MEDICAL ELECTRICITY.

Two months' training in medical electricity are provided at the School of Medical Electricity, 106 Cromwell Road, S.W., of which Mrs. Coghill Hawkes, M.D., is principal, in four courses during the year. Each course consists of theoretical and practical classes, which are held alternately in the afternoons and evenings. At the end of each course an examination will be held; the first course began this month. Students holding the certificate for massage from the Incorporated Society of Trained Masseuses may present themselves for the examination in medical electricity held by the Society.

RATES OF SUBSCRIPTION: Twelve months, 6/6; Six months, 4/-; Three months, 2/-, post free to any address in the United Kingdom. Abroad, Twelve months, 8/8; Six months, 5/-; Three months, 2/6, post free.—Address The Subscription Dept., "THE HOSPITAL," The Hospital Building, 28/29 Southampton Street, Strand, London, W.C.

